# Psychological Capital, Self-Advocacy, and Future Orientation among Adults on the Autism Spectrum

**DOI:** 10.1007/s10803-024-06493-1

**Published:** 2024-07-30

**Authors:** Batel Hazan-Liran, Ofra Walter

**Affiliations:** The Interdisciplinary Research Center for Arts and Spirituality: Therapy, Education and Society, Tel Hai College, Kiryat Shmona, Israel

**Keywords:** Autism spectrum disorder, Psychological capital, Future orientation, Self-advocacy

## Abstract

The paper offers an innovative exploration of the mediating role of psychological capital (PsyCap) in the relations between future orientation and self-advocacy among high-functioning adults on the autism spectrum. We posited that PsyCap, a composite of hope, self-efficacy, resilience, and optimism, serves as a crucial mediator of future orientation and self-advocacy. The sample comprised 40 high-functioning adults on the autism spectrum. Future orientation, self-advocacy, and PsyCap were significantly correlated among young adults with ASD. PsyCap was a mediator of the relations between future orientation and self-advocacy. The findings contribute to the understanding of psychological factors influencing self-advocacy and future orientation and have practical implications for interventions aimed at enhancing PsyCap to improve self-advocacy and future orientation in individuals with autism spectrum disorder.

## Introduction

Autism spectrum disorder (ASD), characterized by challenges in social interaction, communication, and behavior, significantly impacts young adults, a crucial developmental phase extending from adolescence into the mid-twenties, pivotal for establishing social relationships, exploring self-identity, and making critical life decisions. Autistic young adults face compound challenges navigating these milestones (Pearlman-Avnion et al., 2017). We argue three interrelated constructs may help them meet these challenges: self-advocacy, psychological capital (PsyCap), and future orientation. Self-advocacy involves effectively representing one’s interests, needs, and rights, necessitating a comprehensive understanding of personal strengths, weaknesses, and rights (Crane et al., 2019). PsyCap encompasses positive psychological states, including self-efficacy, hope, resilience, and optimism, all of which uniquely influence the cognitive and behavioral patterns of ASD populations (Anselmi et al., 2021). Future orientation entails one’s perspective and planning for the future, and as such, is crucial for planning and goal setting. For autistic young adults, future orientation is complex, often marked by uncertainty and anxiety, but it is essential for a successful transition to adulthood (Brunette et al., 2019).

Given the known impacts of ASD on young adults and the importance of these three constructs, we investigated their interrelationships using a unique theoretical model. We posited a positive linkage between future orientation and self-advocacy, mediated by PsyCap, whereby those with a strong future orientation, including persons with ASD, are likely to leverage their PsyCap more effectively, thus enhancing their self-advocacy. While the individual components of PsyCap, self-advocacy, and future orientation have been studied extensively, their combined effect on young adults with ASD has not been thoroughly explored. We aimed to fill this gap by examining how they interact to support the psychological well-being and developmental progress of autistic young adults.

## The Autism Spectrum

The DSM-5 defines autism as a lifelong neurological disorder (American Psychiatric Association, 2013). A diagnosis of ASD includes developmental delays and is characterized by gaps between various functions. The severity of the disorder varies according to the symptom severity and the person’s level of functioning: low, medium, or high. Functioning level is determined by the level of support required for daily life.

The criteria for diagnosing autism involve persistent and significant deficits in two functional categories (American Psychiatric Association, 2013). The first category is difficulty or absence of social reciprocity, i.e., impairments in communication and social interaction, manifested in three domains: deficits in emotional social reciprocity, impairment in verbal communication used for social interaction, and deficits in developing and maintaining relationships appropriate to the level of development. The first two domains indicate difficulty in understanding social codes and non-verbal communication, and the third indicates difficulties in adapting behavior in a social context. The second functional category involves repetitive behaviors and restricted interests in at least two of the following areas: repetitive speech or motor movements, adherence to routines and verbal or non-verbal rituals, intensely focused and unusual interests, and hyper or hypo-reactivity to sensory input (American Psychiatric Association, 2013).

The navigation of young adulthood (18–25 years) can be markedly different for adults with and without ASD. This period, typified by its potential for growth, exploration, and the opening of new opportunities, is important for the integration of adult-appropriate behaviors and experiences (Anderson et al., 2021). As such, it represents a significant opportunity to develop meaningful social relationships in new settings, such as higher education and professional environments (Orsmond et al., 2013).

While typically developing individuals are expected to gradually progress towards independent living within the community, adults with ASD often face a discordance between their chronological age and emotional or functional maturity. This gap can lead to various challenges in transitioning to independent living, impacting their ability to handle adult responsibilities and roles (Vortman-Shoham & Kenny, 2019). If adults with ASD cannot successfully transition, the social and functional difficulties experienced in childhood may into adulthood; in some cases, they may intensify, leading to more pronounced functional impairments (Orsmond et al., 2013).

### Autism Spectrum and PsyCap

PsyCap is a developmental psychological state, reflecting an individual’s motivational inclination. It includes capabilities that enable adults to overcome barriers in their careers, adapt to new situations, and proactively respond to challenges (Tomlinson, 2017). It comprises four dimensions, partly related to individuals’ beliefs about themselves and partly related to personal resources: self-efficacy, optimism, hope, and resilience (Luthans et al., 2007). These four dimensions guide human behavior, and their combination maximizes human potential as ‘capital’. Self-efficacy is the confidence of an individual in taking on challenging tasks and investing the required effort to succeed in them (Bandura, 1986). Hope is defined as persistence in striving for goals and, if necessary, adjusting strategies to succeed (Luthans et al., 2007). Optimism is defined as attributing positive events to personal, permanent, and pervasive causes and attributing negative events to external, specific, and temporary factors (Carver & Scheier, 2014; Seligman, 1998). Resilience is the ability of an individual to withstand and recover from challenges (Luthans et al., 2007). Adults with ASD are often deficient in one or several of the constructs, hampering their ability to thrive.

Tzemach and Lifshitz (2012) found levels of hope and self-efficacy were significantly higher among individuals with typical development than among those with developmental intellectual disabilities. A study examining the identification of disability and self-efficacy among students on the autism spectrum found less than half expressed a high level of coping with challenges (Shattuck et al., 2014). Developing PsyCap can help individuals with ASD pursue their goals despite inherent challenges (Pesonen et al., 2022).

Resilience is characterized by the ability of an individual to recover from challenges while adapting and adjusting to new realities (Luthans et al., 2007), but individuals with ASD tend to exhibit rigid adherence to routines, express extreme distress due to minor changes, and show difficulties in transitions (American Psychiatric Association, 2013). A research report by Joint Israel revealed that for adults with ASD, any change in the workplace is experienced as a crisis requiring adaptation (Drury, 2015). Developing flexible responses to the changing environment will allow these adults to utilize their skills, live satisfying everyday lives, and develop resilience (Drury, 2015).

A few studies have examined optimism in the context of ASD. Hamilton (2023) investigated the levels of optimism and future orientation among individuals with typical development who had a brother or sister diagnosed on the autism spectrum. The study found they often displayed higher levels of optimism than their peers without such family circumstances and showed a strong future orientation. This suggests that living with a sibling on the autism spectrum may cultivate a perspective that emphasizes hopefulness and proactive planning for the future. Siman-Tov (2016) explored the role of optimism as a resource for parents whose child is diagnosed with ASD. The study highlighted optimism as a crucial coping mechanism that can significantly support parents in navigating the challenges associated with raising a child on the autism spectrum. Optimism appeared to buffer psychological distress and foster adaptive strategies for coping with the unique needs of their children. Although the studies did not focus on individuals with ASD, the findings collectively underscore the multifaceted role of optimism in the broader family context.

PsyCap has been associated with academic motivation, engagement, and achievement (Ononye et al., 2022), but its specific role in self-advocacy and future orientation of young adults with ASD has not been explored. One study considered the experiences, challenges, and needs of autistic transgender young adults (Bruce et al., 2023). We advanced the literature by examining the relationship between future orientation and self-advocacy with PsyCap as a mediator in a sample of young adults with ASD.

### Future Orientation Among Adults with ASD

Future orientation, encompassing an individual’s aspirations, hopes, and fears about the future (Seginer & Lens, 2015), plays a crucial role in the development of autonomy and identity, particularly during adolescence (Zimmer-Gembeck & Collins, 2006). Future orientation involves creating symbolic images of the past, present, and future to construct a future vision. influenced by motivational, cognitive, and behavioral factors aimed at achieving future goals (Nuttin, 1985). For individuals with disabilities, including those with ASD, developing future orientation positively impacts mental and practical functions contributing to better adaptation and improving the quality of life (Schacter et al., 2017).

Autistic young adults may possess distinct perspectives and strategies for envisioning and planning their future. Potential delays in emotional and functional maturity can influence how they perceive and approach future goals, such as developing a career, forming relationships, and achieving independent living (Howlin, 2013). Despite their normal cognitive abilities and potential to specialize in various professional areas, many autistic individuals encounter obstacles integrating into educational and professional fields (Goldfarb et al., 2019; Karkom & Aviv, 2018), leading to a lack of self-confidence, frustration, and low self-esteem (Goldfarb et al., 2019; Knott & Taylor, 2014).

Recognizing and supporting the unique future orientation of these individuals is crucial, as future orientation significantly impacts their capacity to transition into adult roles and responsibilities (Anderson et al., 2016; Taylor & Seltzer, 2011). Research indicates that aspects of PsyCap are crucial for adults with ASD to succeed and align their goals with future aspirations (Luthans et al., 2014; Pesonen et al., 2022). PsyCap plays a significant role in enhancing future orientation by providing a positive reflection on past achievements and prospects (Luthans et al., 2014). It supplies the energy and motivation to aspire and achieve goals, fostering positive expectations and future planning (Saruhan, 2013). As individuals work towards a positive future orientation, their PsyCap is strengthened (Kahana et al., 2012). Ehrlich-Horovitz (2016) found positive correlations between self-efficacy and future orientation in education and careers among teenagers.

Individuals with disabilities often have fewer choice options; this can lower their sense of self-efficacy and hope for the future, leading them to set fewer goals (Malka-Tzemach & Lifshitz, 2012). Studies have shown that a pronounced orientation towards the future helps in the development of autonomy and identity, particularly during adolescence, by providing individuals with a framework for setting goals and making life decisions (Seginer & Lens, 2015; Zimmer-Gembeck & Collins, 2006).

### Self-Advocacy Among Adults with ASD

Self-advocacy involves individuals speaking on their own behalf to represent their wishes and personal interests, protect their rights, and ensure the provision of appropriate services (Atkinson, 2002). It is a central component of the empowerment of individuals with disabilities (Wehmeyer & Little, 2013) and includes three components: self-awareness, rights awareness, and communication (Test et al., 2005). Self-awareness involves understanding one’s strengths and weaknesses within the context of the disability (Stuntzner & Hartley, 2015). Rights awareness pertains to the knowledge of relevant laws and federal policies that regulate the accommodation process for people with disabilities (Mazor, 2013). Communication entails assertive yet non-aggressive interactions with service providers (Test et al., 2005).

The principle of ‘Nothing about us without us’ (Charlton, 1998) emphasizes the necessity for people with disabilities to be part of decision-making processes affecting their status, underscoring the importance of self-representation (Shakespeare, 2006). Service recipients often possess unique knowledge from life experiences that professionals, policymakers, and academics lack (Rutz et al., 2018), but they must be able to disseminate their knowledge. In this context, studies have highlighted the positive impact of self-advocacy skills. One study found students with visual impairments used self-advocacy to manage their impairments and take responsibility for their lives (Hess, 2018).

Self-awareness is crucial for self-advocacy, especially for individuals with ASD. Knowing their diagnosis enables them to present their abilities and challenges accurately, ensuring appropriate services (Tapia, 2023). Early self-understanding before adulthood can reduce conflicts and foster self-definition, impacting self-advocacy, identity, and stress management positively (Kim, 2019). Conversely, concealing a diagnosis may impair the development of self-advocacy skills (Crane et al., 2019).

Self-advocacy can influence future orientation. As such, it can be beneficial in the workplace. A recent study found constructing a narrative of employability was linked to self-advocacy (Pesonen et al., 2022). However, research on the relations between self-advocacy and future orientation among adults with ASD is limited. If they recognize their strengths, they can align them with future goals, but a lack of self-advocacy may present a barrier to planning and imagining the future in various life domains. Research in multiple countries (Finland, England, France, and the Netherlands) has shown that recognizing one’s strengths and aligning them with career goals enhances career development (Pesonen et al., 2022). One study found the provision of peer support spaces for adults on the autism spectrum enabled these adults to share their feelings and assisted them in developing self-advocacy skills, leading to a positive change in self-perception (Goldfarb et al., 2019). However, little research addresses the relations between self-advocacy and future orientation among adults with ASD.

PsyCap and self-advocacy skills together are vital for psychological well-being (To, 2007). Developing self-advocacy strengthens self-efficacy and resilience, improving quality of life (Hirschberg, 2018; Sedillo-Hamann, 2021). The literature on self-advocacy and PsyCap components (hope, welf-efficacy, resilience, optimism) indicates that enhancing coping skills and resilience can develop self-advocacy in people with disabilities (Stuntzner & Hartley, 2015). In autism, this interplay enables the effective navigation of social, educational, and vocational challenges (Tapia, 2023). For individuals with ASD, the interplay between self-advocacy and PsyCap can be particularly influential. Concurrently, the components of PsyCap facilitate the resilience and optimism needed to pursue these goals, despite the challenges inherent to their condition. This synergy is crucial in contexts such as vocational training and integration into the workforce, where self-advocacy and PsyCap collectively enhance the potential for success (Luthans et al., 2007a; Raz, 2019).

### The Present Study

We addressed the gap in literature by examining the relations between self-advocacy, PsyCap, and future orientation among young adults with autism. The sample comprised individuals diagnosed on the high-functioning end of the autism spectrum who were receiving support from a center for young adults diagnosed with autism. We specifically asked how PsyCap mediates the relations between future orientation and self-advocacy. By so doing, we hoped to provide new insights into the psychological mechanisms that support the transition to adulthood for individuals with ASD, with practical implications for tailored interventions and support systems aimed at enhancing the psychological well-being and developmental progress of autistic young adults.

Our first objective was to look for positive relations between PsyCap, future orientation, and self-advocacy. We hypothesized that a higher degree of future orientation would correlate with increased levels of PsyCap and self-advocacy, and vice versa. This hypothesis was grounded in the assumption that a more developed future orientation helps individuals define their aspirations and goals. We expected this would be linked to stronger PsyCap and to more effective self-advocacy, thus facilitating how individuals mediate themselves and their objectives within their environment.

Our second objective was to explore whether within this population, PsyCap mediates relations between future orientation and self-advocacy. We hypothesized that individuals with ASD with a higher future orientation would exhibit a higher level of PsyCap, and this, in turn, would enhance their self-advocacy capabilities. This hypothesis was based on the premise that an optimistic vision of the future, coupled with the ability to envisage desirable goals, may amplify PsyCap. This enhancement may assist in actualizing personal potential by converting tasks from potential to action through intrinsic motivation. PsyCap is thought to aid in optimistic thinking and in the overcoming of obstacles on the path to achieving one’s goals. The capacity for hope is instrumental in goal setting and may assist in self-advocacy. Therefore, we anticipated finding enhanced self-advocacy as well.

## Method

We employed a quantitative research method to investigate the relations between self-advocacy, PsyCap, and future orientation among autistic young adults.

### Participants

After receiving ethics clearance for the research from the institutional ethics committee, we recruited 40 participants diagnosed with autism who were affiliated with a support organization designed specifically for this population. The sample comprised 28 males (70%) and 12 females (30%), aged 18–30 years (M = 23.6, SD = 3.9). The age at which participants were diagnosed ranged from 1 to 29 years (M = 11.82, SD = 7.46). They had been associated with the support organization for an average of 20 months (from 1 to 72 months, SD = 18.62). All were single and had a current diagnosis classifying them as high-functioning. A valid diagnosis was a mandatory requirement set by the support organization as a condition for inclusion in its programs. The official diagnoses of all participants had been presented to the organization’s social worker and were filed in their personal records for documentation and monitoring purposes.

The support organization constructs flexible service packages for all adults with autism, based on the life areas for which they wish to receive support. One program is a preparatory program for independent living; this is a residential program aimed at facilitating a training and experiential period for adults with ASD, in preparation for autonomous life in the community (*n* = 11, 27.5%). Employment programs offer occupational guidance, with the goal of helping all those with ASD realize their employment potential; program participants receive a personalized plan, integrating tools, training, and guidance tailored to their current stage, desires, and readiness (*n* = 11, 27.5%). A housing program helps adults with ASD rent apartments autonomously and independently in the community (*n* = 15, 37.5%). A community living program focuses on challenging behavioral issues; it emphasizes creating a homey, safe, and inclusive environment and reducing stress situations and risky behaviors, while providing support and assistance in various life aspects (*n* = 8, 20%). Finally, an education program supports individuals with ASD in higher education, facilitating academic learning by making it accessible and assisting students until the completion of their degree/certificate (*n* = 4, 10%). Some of our study participants were involved in multiple programs simultaneously.

Participants responded to a questionnaire, administered as an online survey using the online Qualtrics questionnaire, in the presence of a research assistant who worked at the support organization and was familiar with the methods of working with the population of interest. The research assistant was present during the completion of the questionnaires to provide linguistic clarifications when needed. Participants were informed of the objectives and contributions of the study before beginning the online survey and signing the consent form. Participation was voluntary with no monetary compensation.

### Instruments

We used four questionnaires to test the research hypotheses.

#### Demographic Questionnaire

The demographic questionnaire included several sections, each focusing on a different aspect of the required information:

##### Personal and Demographic Information

This section included questions on age, gender, and year of birth.

##### Diagnosis Details

This part of the questionnaire addressed the year of autism diagnosis and the key findings leading to the diagnosis. The questions were structured to gather detailed information about the diagnostic process, including any significant milestones or characteristics that were pivotal in identifying the condition.

##### Integration Framework

This section required information about the type of educational or therapeutic setting with which the participant was involved at the support organization. It aimed to collect details on the nature of the support environment, for example, a specialized educational program, a therapeutic setting, or another form of assistance.

### Psychological Capital Questionnaire (PCQ)

The PCQ is a validated instrument designed to measure four core positive psychological capacities: self-efficacy, hope, resilience, and optimism. The original PCQ-24, developed by Luthans et al. (2007), has been widely used in organizational psychology. To streamline the assessment, Avey et al. (2011) developed a 12-item version (PCQ-12), maintaining strong psychometric properties. The PCQ-12 comprises 12 items rated on a 5-point Likert scale, from 1 (strongly disagree) to 5 (strongly agree). As our participants were individuals with ASD and most were not employed, we adapted the phrasing to reflect their involvement in the support organization.

The PCQ-12 has demonstrated robust internal consistency, with Cronbach’s alpha coefficients ranging from 0.70 to 0.85 across various studies (e.g., Avey et al., 2011; Dawkins et al., 2013). Factor analyses consistently support the four-factor structure, confirming the distinctiveness of each psychological capacity (Dawkins et al., 2013; Lorenz et al., 2016). Validity assessments have shown the PCQ-12 possesses good convergent validity, correlating positively with other psychological constructs like self-efficacy, hope, and optimism (Avey et al., 2011a; Rego et al., 2012), and good discriminant validity, with low correlations with unrelated constructs. Criterion-related validity is evidenced by positive associations with desirable outcomes such as job satisfaction and performance, and negative associations with job stress and turnover intentions (Avey et al., 2010; Luthans et al., 2008). These findings underscore the PCQ-12’s reliability and validity as a concise measure of PsyCap in diverse contexts.

Table 1 shows the division of the subscales, including their reliability in this study. Note that although the questionnaire comprises several factors, it is commonly treated and analyzed as a single measure, wherein the whole is greater than the sum of its parts (Avey et al., 2011a; Luthans & Youssef-Morgan, 2017).

**Table 1:** Division and reliability of PCQ subscales

Scales	α	Sample item
Self-efficacy	0.89	I feel confident analyzing a long-term problem to find a solution.
Hope	0.74	There are lots of ways around any problem.
Optimism	0.71	When things are uncertain for me at the association, I usually expect the best.
Resilience	0.76	I can get through difficult times at the association because I’ve experienced difficulty before.
Overall	0.90	

### *Self-Advocacy Questionnaire*

The Self-Advocacy Questionnaire, initially developed as part of the ‘My Future My Plan’ program (Wurzburg et al., 2003) to aid transition planning for individuals with disabilities, was adapted and translated by Katsir (2020) to explore self-advocacy among adults with developmental intellectual disabilities. This 30-item instrument is a reliable and valid tool for assessing self-advocacy in diverse contexts (Katsir, 2020; Wurzburg et al., 2003). It assesses various dimensions of self-advocacy, including knowledge of rights and community services, communication and self-direction, assertiveness and initiative, and self-awareness. Items are rated on a 5-point Likert scale from 1 (strongly disagree) to 5 (strongly agree), with higher scores indicating greater self-advocacy. It is often treated as a single composite measure, providing a holistic understanding of self-advocacy skills (Mohar, 2010; Zuber & Webber, 2019). Higher scores are associated with positive outcomes such as increased community participation and improved quality of life, underscoring its criterion-related validity. Psychometric evaluations have demonstrated the questionnaire’s robust internal consistency, with Cronbach’s alpha coefficients ranging from 0.75 to 0.88 across different subscales (Katsir, 2020). Factor analyses support its multidimensional structure, and validity assessments reveal strong convergent and discriminant validity, correlating well with related constructs like self-determination and empowerment while maintaining low correlations with unrelated constructs. Table 2 shows the division of the subscales, including their reliability in this study.

**Table 2:** Division and reliability of self-advocacy subscales

Scales	α	Sample item
Knowledge of rights and services	0.86	I frequently spend time in community places.
Communication and self-direction	0.82	I express my needs and desires.
Assertiveness and initiative	0.88	I ask for help from others.
Awareness and self-acceptance	0.86	I know what my skills are.
Overall	0.95	

### Future Orientation Questionnaire

The Future Orientation Questionnaire, developed by Seginer et al. (1991), is designed to assess the motivational, cognitive, and behavioral aspects of future orientation across various life domains. For the purposes of our study, we omitted the first part of the questionnaire addressing hopes and fears about the future. Our adapted version consisted of 27 items, divided into two dimensions: work and study. Respondents rate each item on a 3-point scale, where 1 signifies a non-active future orientation (not at all) and 3 signifies a very active future orientation (to a great extent). Higher scores indicate a greater level of future orientation. The questionnaire is often analyzed as a unified measure to provide a comprehensive assessment of future orientation, despite the presence of multiple subscales (Seginer, 2009). Psychometric evaluations of the original and adapted versions have shown good internal consistency, with Cronbach’s alpha coefficients typically exceeding 0.70 for the subscales (Seginer, 2003; Seginer et al., 1991). Factor analyses support the questionnaire’s two-dimensional structure, confirming the distinctiveness of the work and study domains. Validity assessments have demonstrated strong convergent validity, with significant correlations between future orientation and related constructs such as goal-setting and planning behaviors. The questionnaire has also shown good discriminant validity, with low correlations with unrelated psychological constructs. Criterion-related validity is evidenced by positive associations between high future orientation scores and favorable outcomes such as academic achievement and career planning (Nurmi, 1991; Poole, 2001). Table 3 shows the division of the subscales, including their reliability in this study.

**Table 3:** Division and reliability of future orientation subscales

Scales	α	Sample item
Work	0.89	How frequently do you think about workplaces where you would like to work?
Study	0.92	What importance will studies have in your future life?
Overall	0.92	

### Research Procedure Overview

After obtaining approval from the institutional ethics committee, we administered a digital questionnaire created using Qualtrics and made accessible on personal computers and smartphones. The study began with an introduction to the research aims, participant anonymity, and data confidentiality. Participants provided informed consent prior to questionnaire completion and had access to the researcher’s email for inquiries. The questionnaires were distributed primarily by the staff of the support organization, targeting persons enrolled in its programs. All those affiliated with the organization were eligible to respond to the survey. Detailed instructions were provided for each questionnaire section, with an option for verbal assistance. Some participants received assistance from counselors to complete the questionnaire.

The survey sequence included an introductory explanation, consent form, demographic information, PsyCap assessment, a self-advocacy questionnaire, and a section on future orientation. The process typically took about 10 min. Data were analyzed using IBM SPSS Statistics 27 software. Pearson’s test was applied to examine relations between research variables.

### Community Involvement Statement

Significant community involvement was a vital part of the research. In the literature review, methodology development, and data analysis phases, we consulted key actors at the support organization. Following the determination of the findings, we had further discussions with these actors to formulate a plan in alignment with these findings. It is noteworthy that a program for the development of PsyCap and self-advocacy skills has already been initiated by the support organization, underscoring the importance of the research findings.

## Results

### Descriptive Data

The descriptive data are shown in Table 4.

**Table 4:** Questionnaire averages for future orientation, psycap, and self-advocacy

Variables	Subscale	Mean (SD)
**Self-Advocacy**	Knowledge of rights and services	3.43 (0.72)
	Communication and self-direction	3.81 (0.61)
	Assertiveness and initiative	3.55 (0.86)
	Awareness and self-acceptance	3.61 (0.77)
	Total	3.59 (0.67)
**PsyCap**	Self-efficacy	3.46 (1.19)
	Hope	3.43 (0.84)
	Optimism	3.46 (0.98)
	Resilience	3.27 (0.89)
	Total	3.40 (0.82)
**Future Orientation**	Work	2.18 (0.40)
	Study	2.02 (0.55)
	Total	2.12 (0.40)

### Confirmatory Factor Analysis

Since the research questionnaires were modified to align with our objectives, we conducted confirmatory factor analysis (CFA) to ensure the alterations did not compromise the internal consistency of each questionnaire. We used Amos 28 edition to validate the relations between the latent variables (future orientation, self-advocacy, PsyCap) and the observed variables (item ratings on a specific questionnaire). CFA is a used to determine whether the collected data align with a hypothesized model, comprising a latent variable and its corresponding observed indicators. It evaluates the strength of the correlations between these variables, with acceptable indicator loadings being λ > 0.25 (Schreiber et al., 2006; Suhr, 2006). Table 5 presents the loadings and explained variances for the study variables.

**Table 5:** CFA of the latent variable psycap and academic adjustment

Variables (Latent)	Subscale (latent)	Load (λ)	*R* ^2^
**Future Orientation**	Work	0.93	0.87
	Study	0.53	0.28
**PsyCap**	Self-efficacy	0.74	0.55
	Hope	0.90	0.81
	Optimism	0.73	0.54
	Resilience	0.73	0.53
**Self-Advocacy**	Knowledge of rights and services	0.82	0.67
	Communication and self-direction	0.89	0.78
	Assertiveness and initiative	0.96	0.93
	Awareness and self-acceptance	0.93	0.86

We used three indices to determine how well the hypothesized models fit the data. The first two indices, the comparative fit index (CFI) and the Tucker-Lewis Index (TLI), range from 0 to 1, with higher values indicating better model fit. The third index, the root mean square error of approximation (RMSEA), ranges from 0.0 to 0.1, with lower values indicating better model fit. A satisfactory model fit is indicated by CFI and TLI values of ≥ 0.90 and RMSEA of ≤ 0.06 (Suhr, 2006). The fit for the CFA model was excellent, with CFI = 0.97, TLI = 0.96, and RMSEA = 0.08.

### Hypothesis 1: Correlation of Future Orientation, Self-Advocacy, and PsyCap

Our first hypothesis was that future orientation, self-advocacy, and PsyCap would be significantly correlated among young adults with ASD. As Table 6 shows, they were positively and statistically correlated, thus fully corroborating Hypothesis 1. More specifically, as the levels of future orientation increased, so did the levels of self-advocacy and PsyCap.

**Table 6:** Spearman correlations between future orientation, PsyCap, and self-advocacy

Scales	1	2	3
**1. Self-Advocacy**	1	-	-
**2. PsyCap**	.84***	1	-
**3. Future Orientation**	.57***	.59***	1

*** *p* < .001, ** *p* < .01, * *p* < .05

### Hypothesis 2: PsyCap as Mediator of Future Orientation and Self-Advocacy

Our second hypothesis was that PsyCap would mediate the relations between future orientation and self-advocacy. The PROCESS procedure, model 4, developed by Andrew F. Hayes, is a statistical method used for mediation, moderation, and conditional process analysis. It allows researchers to explore the direct and indirect effects of variables within their models (Hayes, 2018). We used the PROCESS procedure to examine the second hypothesis, with future orientation as the independent variable, self-advocacy as the dependent variable, and PsyCap as a mediator. Results showed future orientation positively predicted PsyCap, B = 1.201, S.E.=0.270, CI; 0.654–1.749. PsyCap, in turn, positively predicted self-advocacy, B = 0.627, S.E.=0.090, CI; 0.444–0.810. The direct effect of future orientation on self-advocacy was statistically insignificant, B = 0.210, S.E.=0.185, CI; -0.165–0.586.

**Fig. 1:**
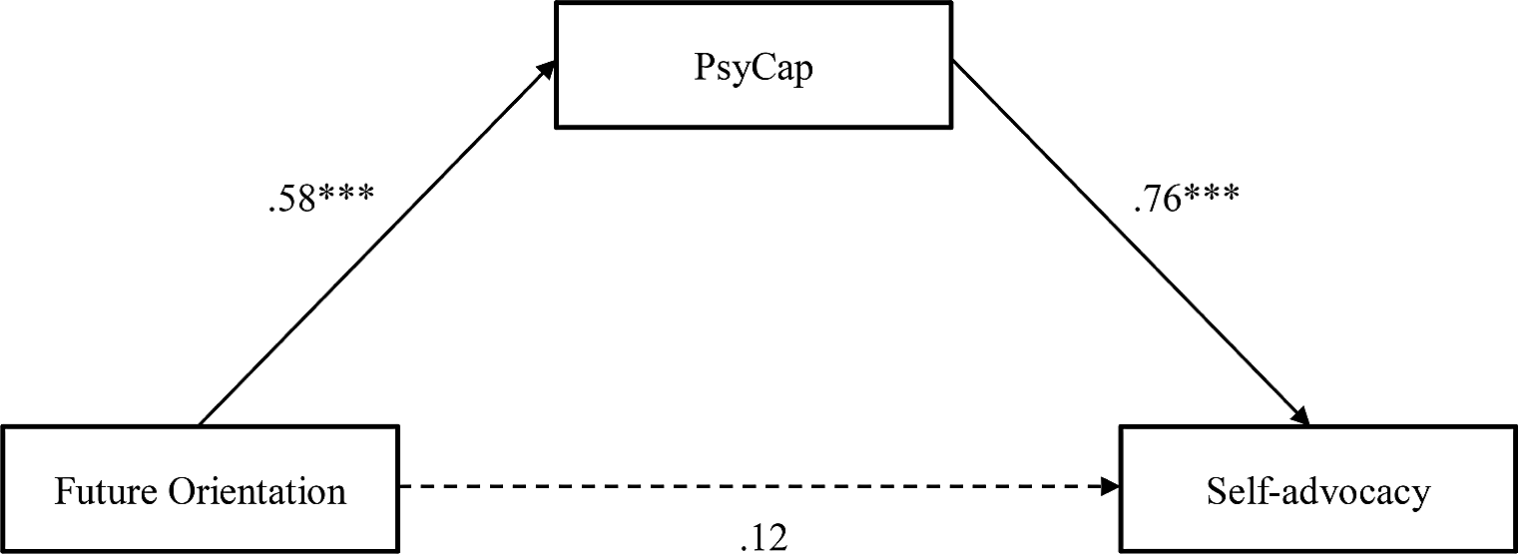
Relations between future orientation and self-advocacy via psycap as a mediator (reporting beta correlation)

To test the significance of the indirect effect, we employed a bootstrapping technique, utilising 5,000 resamples, to generate 95% confidence intervals (CIs). Indirect effects in which zero is not included in the 95% CI indicate a significant effect at α < 0.05. Tests of the indirect effect of future orientation on self-advocacy via PsyCap were significant, B = 0.754, S.E.= 0.217, CI; 0.294–1.152. The mediation analysis showed higher future orientation was associated with higher PsyCap, and higher PsyCap was associated with higher self-advocacy (Fig. 1). While the total effect of future orientation on self-advocacy was significant, the direct effect was not, indicating PsyCap fully mediated the relations between future orientation and self-advocacy.

## Discussion

We examined the relations between PsyCap, future orientation, and self-advocacy in a sample of high-functioning adults with ASD. Hypothesis 1 argued an enhanced future orientation would be positively correlated with elevated levels of PsyCap and self-advocacy. This hypothesis was based on the notion that a distinct vision of the future helps individuals define their aspirations and goals, thereby reinforcing their psychological resources and enhancing their self-advocacy skills. Our findings supported this hypothesis. We found significant correlations between future orientation, PsyCap, and self-advocacy, consistent with prior research (Goldfarb et al., 2019; Karkum & Aviv, 2018).

Individuals with ASD, particularly adults, often encounter challenges when developing a future orientation. They may have difficulties in areas such as education and employment, even though they have the cognitive abilities and potential to excel (Goldfarb et al., 2018). These challenges are compounded by the fact that only a minority actively pursue educational opportunities, a critical factor in shaping future orientation. Luthans et al. (2014) argued PsyCap plays a crucial role in fostering a positive outlook on future achievements. This, in turn, enhances future orientation. PsyCap allows individuals to reflect positively on past achievements and provides the motivation and energy they need to pursue and attain goals, fostering positive expectations and planning for the future (Saruhan, 2013). Kahana et al. (2012) found that as individuals develop a more positive future orientation, the dimensions of their PsyCap are strengthened.

Our finding of a strong correlation between PsyCap, self-advocacy, and future orientation is particularly notable. Ehrlich-Horovitz (2016) identified strong correlations between self-efficacy, a component of PsyCap, and future orientation in the context of education and career aspirations among teenagers involved with organizations. Together, these findings underscore the importance of self-advocacy skills in enabling individuals, particularly those with ASD, to feel in control of and hopeful about their future.

The second hypothesis was that individuals with ASD with a pronounced future orientation would demonstrate elevated levels of PsyCap, and this, in turn, would amplify their self-advocacy. Our fundings supported this hypothesis by revealing a significant indirect relationship between future orientation and self-advocacy, mediated by PsyCap. This finding is intriguing, as it suggests the direct link between future orientation and self-advocacy is less pronounced in the presence of PsyCap. Instead, it is the indirect pathway through PsyCap that mainly influences self-advocacy skills. This suggests PsyCap plays a crucial, intermediary role in how individuals with ASD translate their future-oriented thinking into practical self-advocacy actions. The concept of PsyCap is critical in this context. Its components of hope, resilience, optimism, and self-efficacy can provide individuals with the psychological resources they need to not only envision a positive future but also to take proactive steps towards achieving it. Others have similarly underscored the importance of PsyCap in various aspects of life and work outcomes (Avey et al., 2011b; Luthans et al., 2007b). For individuals with ASD, these psychological resources are particularly important, as they have unique challenges in developing self-advocacy and autonomy.

Our finding of indirect relations between future orientation and self-advocacy, mediated by PsyCap, emphasizes the need for interventions that foster psychological resilience and optimism in individuals with ASD. Enhancing PsyCap could serve as a pivotal strategy in enabling them to translate their future goals into self-advocacy actions. This type of approach could possibly bridge the gap between the cognitive aspect of planning and the practical aspect of advocating for oneself.

Moreover, our finding of non-significant direct relations between future orientation and self-advocacy in the presence of PsyCap suggests that simply having future-oriented thoughts is not sufficient for effective self-advocacy among individuals with ASD. Instead, their psychological resources must be bolstered to allow them to more effectively navigate and articulate their personal needs and aspirations. This highlights the multifaceted nature of self-advocacy and the importance of a holistic approach in supporting individuals with ASD. The challenges in building future orientation among adults with ASD, often stemming from a deficit in self-advocacy skills, highlight the need for targeted interventions. Our findings suggest interventions aimed at enhancing PsyCap and self-advocacy may be effective in activating and strengthening future orientation capabilities in adults with ASD. This aligns with the broader understanding of the role of psychological resources in facilitating better outcomes in life and work, as suggested by Luthans et al. (2007) and Avey et al. (2011)

### Implications

This study has important implications for the understanding of psychological mechanisms in high-functioning individuals with ASD. The significant correlation between future orientation, PsyCap, and self-advocacy underscores the critical role of PsyCap, with its elements of hope, resilience, optimism, and self-efficacy, in fostering a future-oriented mindset and self-advocacy skills. The finding that the relationship between future orientation and self-advocacy is mediated by PsyCap suggests a new area of focus for interventions. Enhancing PsyCap in individuals with ASD could be a key strategy in boosting their ability to envision a positive future and empowering them to actively advocate for their needs and goals.

#### Limitations and Future Research

Admittedly, the study had some limitations. First, the study’s reliance on self-reported measures may have introduced biases or inaccuracies. Recruiting a larger sample and incorporating a mix of qualitative and quantitative methods, including observations and third-party assessments, could yield a more comprehensive understanding of the study constructs. Second, the focus on high-functioning individuals with ASD could limit the generalizability of the findings to the broader ASD population. Individuals with different levels of functioning may exhibit varying degrees of PsyCap and self-advocacy skills, suggesting a need for research across a broader spectrum of ASD.

PsyCap is regarded as relatively malleable and amenable to development; in other words, it is not static but open to change. It encompasses positive resources that can be enhanced through short-term interventions (Luthans et al., 2006, 2008; Luthans, Avolio Luthans et al., 2007a, b). Research is needed to develop targeted interventions to enhance PsyCap and self-advocacy in individuals with ASD. Such work could explore the effectiveness of different types of interventions, including educational programs, psychological therapies, and support groups, in boosting future orientation and self-advocacy skills in this population.

## Conclusion

The study illuminates the interconnectedness of future orientation, PsyCap, and self-advocacy in individuals with ASD. The results confirm that a stronger future orientation correlates with increased PsyCap and enhanced self-advocacy skills in this population. Notably, they reveal the crucial mediating role of PsyCap in linking future orientation to self-advocacy, highlighting the importance of psychological resources like hope, resilience, self-efficacy, and optimism. The study contributes to the growing body of literature on PsyCap and self-advocacy in individuals with ASD, offering promising directions for future research and intervention development.
